# The intracellular proteome of African swine fever virus

**DOI:** 10.1038/s41598-018-32985-z

**Published:** 2018-10-02

**Authors:** Catharina Keßler, Jan H. Forth, Günther M. Keil, Thomas C. Mettenleiter, Sandra Blome, Axel Karger

**Affiliations:** 1 Institute of Molecular Virology and Cell Biology, Friedrich-Loeffler-Institut, Federal Research Institute for Animal Health, Südufer 10, 17493 Greifswald-Insel Riems, Germany; 2 Institute of Infectology, Friedrich-Loeffler-Institut, Federal Research Institute for Animal Health, Südufer 10, 17493 Greifswald-Insel Riems, Germany; 3 Institute of Diagnostic Virology, Friedrich-Loeffler-Institut, Federal Research Institute for Animal Health, Südufer 10, 17493 Greifswald-Insel Riems, Germany

## Abstract

African swine fever (ASF) is a viral disease that affects members of the *Suidae* family such as African bush pigs, warthogs, but also domestic pigs, and wild boar. It is transmitted by direct contact of naïve with infected animals, by soft ticks of the *Ornithodoros* genus, or indirectly by movement of infected animals, improper disposal of contaminated animal products or other sources related to human activity. The recent spread of ASF into Eastern and Central European countries is currently threatening the European pig industry. The situation is aggravated as to-date no efficient vaccine is available. African swine fever virus (ASFV) is a large enveloped ds DNA-virus encoding at least 150 open reading frames. Many of the deduced gene products have not been described, less functionally characterized. We have analysed ASFV protein expression in three susceptible mammalian cell lines representing a susceptible host (wild boar) and two non-susceptible species (human and green monkey) by mass spectrometry and provide first evidence for the expression of 23 so far uncharacterized ASFV ORFs. Expression levels of several newly identified ASFV proteins were remarkably high indicating importance in the viral replication cycle. Moreover, expression profiles of ASFV proteins in the three cell lines differed markedly.

## Introduction

In the past decades, African swine fever (ASF) has repeatedly been introduced from sub-Saharan Africa to Europe. The most recent introduction in 2007 had its origin in Eastern Africa and started in Georgia by improper disposal of contaminated food in the vicinity of Poti harbour^1^ from where it spread across the Caucasus and the Russian Federation to reach the eastern border of the European Union in early 2014. The virus is now enzootic in Estonia, Latvia, Lithuania, the Ukraine, Belarus, and Poland and has spread to other European countries, e.g. the Czech Republic and Romania as well. The westward expansion of the disease is a serious threat to the pig industry in central and western European countries^2,3^. In spite of this increasingly threatening situation and many efforts, no effective vaccine has been developed to-date^4^.

African swine fever virus (ASFV), the causative agent of African swine fever, is the only member of the family *Asfarviridae* in the nucleocytoplasmic large DNA virus superfamily^5,6^. In different isolates, the size of the double-stranded DNA genome ranges from 170 kbp to 190 kbp, and contains over 150 predicted open reading frames (ORFs). In addition to the proteins that are required for basic viral functions like replication and morphogenesis, numerous ASFV proteins modulate cellular pathways like apoptosis or innate immunity, accounting for the high complexity of virus-host interactions^7^. The fact that ASFV can replicate in swine as well as in its arthropod vector, soft ticks of the *Ornithodoros* genus, may contribute to the complexity of ASFV protein expression. In addition to the 54 structural proteins that form the complex ASF virion^8^, a number of non-structural proteins are also encoded. However, many of them are completely uncharacterized and lack any evidence for expression in cell culture or in infected animals. They frequently belong to multi gene families (MGF) which are known to influence cell tropism and host range of ASFV^9–11^.

The intent of this study was to identify the intracellular proteome of ASFV, to provide a comprehensive catalogue of ASFV proteins expressed in mammalian cell culture and to identify a core set of ASFV proteins required to support infection of permissive cultured mammalian cells, but also to elucidate potential host-specific differences in the expression profiles. Since immunologic reagents for specific detection are lacking for most ASFV proteins, we have applied mass spectrometry (MS) which, as an ‘open-view’ tool, allows the detection of proteins by identification of peptides deduced from the viral genomic sequence. A GFP-expressing thymidine kinase (TK) negative recombinant of ASFV strain OURT 88/3 (OURT 88/3-ΔTK-GFP) was used for infection. OURT 88/3 is a naturally attenuated non-hemadsorbing field strain isolated from *Ornithodoros erraticus* ticks in Portugal^12^ which is considered for use as vaccine as it has shown the ability to protect pigs against a challenge with virulent ASFV strain OURT 88/1^12–14^. Three susceptible cell lines were selected for proteome analysis after infection. WSL-HP is a lung cell line from wild boar, a natural host of ASVF, while HEK 293 cells originate from humans which are not susceptible to infection. Vero cells were included, since they have been extensively used for infection experiments with ASFV in the past.

## Results and Discussion

### MS study design

**Figure 1:**
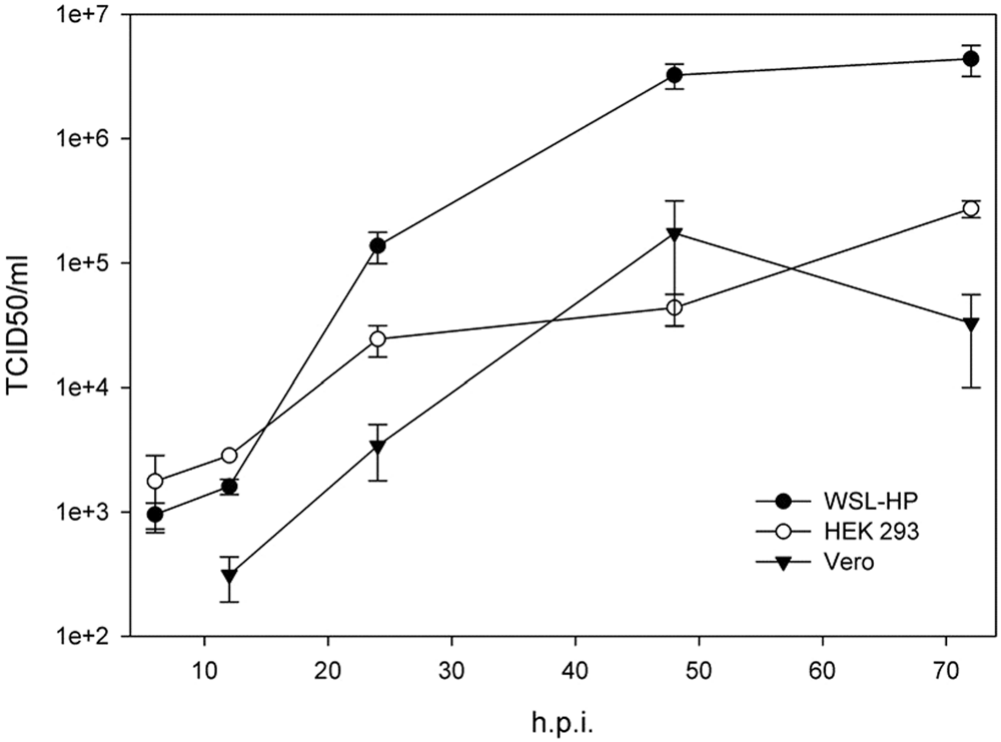
ASFV OURT 88/3-ΔTK-GFP productively infects WSL-HP, Vero, and HEK 293 cells. After inoculation of WSL-HP, Vero, and HEK 293 cells with OURT 88/3-ΔTK-GFP, supernatants were collected at the times indicated and titers of the progeny virus were determined on WSL-HP cells. The *in-vitro* growth kinetics show that ASFV OURT 88/3-dTK-GFP replicates well on all three cell lines, although final titers on Vero and HEK 293 cells are over tenfold lower than on WSL-HP cells.

**Figure 2:**
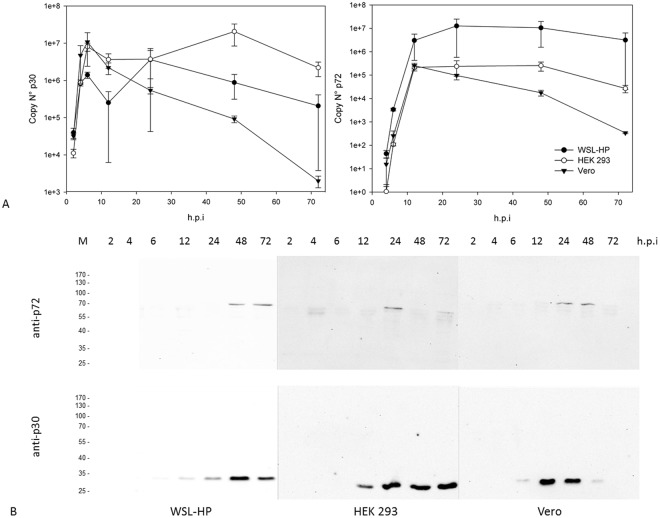
*In-vitro* expression kinetics of OURT 88/3 proteins p30 and p72. Cells were infected with ASFV OURT 88/3-ΔTK-GFP and harvested at the times indicated. Panel A shows the quantitation of the p30 (left) and p72 (right) expression by RT-qPCR and panel B an immunoblot analysis, respectively. p30 is expressed with early, p72 with late kinetics, time points are given in hours after infection (h.p.i.). The means of 2 independent experiments are shown. At the time of harvest for MS, p72 is expressed in all three cell lines.

The three cell lines used in this study were fully susceptible to infection with the recombinant ASFV OURT 88/3-ΔTK-GFP expressing GFP under the control of the late ASFV p72 promotor^15–17^. As shown in Fig. 1 the virus replicated in all three cell lines, although final titers on Vero and HEK 293 were 20-fold and 16-fold lower than on WSL-HP cells, respectively. Before preparing samples for the MS analysis the infection conditions were adjusted to ensure that completely infected cell monolayers were used for the preparation of the protein extracts in order to achieve high sensitivity for the detection of ASFV proteins and to enable the MS based quantitative comparison of ASFV protein expression between the different cells. Cell monolayers were inoculated with ASFV OURT 88/3-ΔTK-GFP and GFP fluorescence, indicating the entry into the late phase of infection, was monitored over time. After 24 h (HEK 293 and Vero) and after 48 h (WSL-HP) the cell monolayers were entirely positive for GFP fluorescence. Immunoblot and RT-qPCR analyses targeting structural proteins p30, expressed with early kinetics^18,19^, and p72, expressed with late kinetics^15,16^, confirmed that the cell lines were in the late phase of infection (Fig. 2) at the given time points which were then chosen to harvest the cells for the MS analysis.

For the proteome analysis of the cell extracts a shotgun approach was applied using a mass spectrometric platform combining nano liquid chromatography (nLC) and matrix-assisted laser desorption/ionisation tandem time-of-flight (MALDI-TOF/TOF) MS. In short, total protein extracts of the three cell lines were prepared at the designated time points, proteins were digested into peptides, separated by nLC and analysed on the MALDI-TOF/TOF instrument. The protein composition of the extracts was then determined by a query of the spectra using a sequence database containing host and virus proteins. To increase sensitivity and improve sequence coverages of ASFV proteins in the mass spectrometric analysis, two replicates of each of the three infected cell lines were digested with three different proteases with complementary specificity (trypsin, chymotrypsin and Glu-C). The choice of the proteases based on an *in-silico* comparison of the physical properties (molecular weight, isoelectric point, pI, and the Grand Average of Hydropathy, GRAVY^20^) of ASFV and *Sus scrofa* proteins which had indicated that the viral proteins were, on average, smaller (37.7 kDa vs.50.5 kDa), more basic (pI 7.81 vs. 7.33), and more hydrophobic (GRAVY −0.21 vs. −031) than the *Sus scrofa* proteins (Supplementary Fig. S1). The results of the six nLC-MALDI-TOF/TOF MS runs for every cell line were compiled into a single result file for the calculation of the identification scores, sequence coverages and the number of peptides identified in every protein. A comprehensive representation of the MS data is provided as Supplementary data, Tables S1 and S2.

Classification of ASFV proteins into structural, non-structural and uncharacterized proteins followed a recent review^6^. Additionally, the literature was searched for any evidence for the expression of ASFV RNAs or proteins and the references were added to the column ‘Ref’ in Supplementary Table S3. To the best of our knowledge, we demonstrate the existence of 23 ASFV proteins, for which no evidence of expression was available so far.

### Qualitative proteome analysis

**Figure 3:**
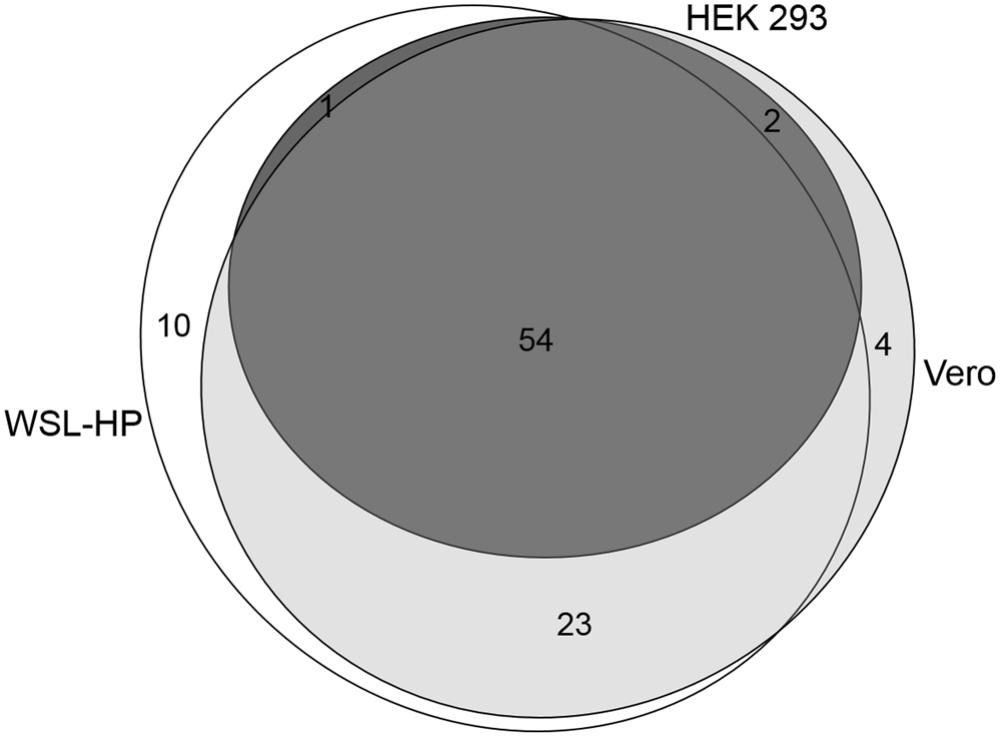
Comparison of the ASFV proteins expressed in WSL-HP, HEK 293 and Vero cells. In total, 94 ASFV proteins were identified by MS, 54 of them in all three cell lines. Of the remaining 40, 23 were shared by Vero (light grey) and WSL-HP (white). Ten, and four proteins were exclusively expressed in WSL-HP and in Vero cells, respectively.

The results of the qualitative MS analyses are summarized in Fig. 3. In total, for 94 of the 157 OURT 88/3 ORFs the corresponding proteins could be identified. Thirty-seven of these identifications confirm known proteins and for further 34 evidence for transcription, but not for the existence of the corresponding protein, can be found in the literature. These include 6 out of the 32 members of the MGF genes present in OURT 88/3. For 23 of the identified proteins we have not found any evidence for expression of the corresponding gene in the current literature, neither for the presence of a transcript, nor of a protein. The detailed results obtained for all 94 identified ASFV proteins in the three different cell lines are presented in Supplementary Table S3.

Expression profiles of ASFV specific proteins differed markedly between the cell lines (Fig. 3). Fifty-four proteins were detected in all three cells which may represent a core set of ASFV proteins required to maintain the late phase of infection mammalian cell cultures. Ten proteins were exclusively detected in WSL-HP cells, while 4 more were exclusively identified in Vero cells. Twenty-three proteins were shared between WSL-HP and Vero cells but were undetectable in HEK 293 cells.

The observed differences were obviously not due to technical issues, as the low number of 57 identified viral proteins in HEK 293 cells was in contrast to the high number of 1,251 identified host cell proteins (vs. 1,194 in Vero and 969 in WSL-HP) and may reflect a limitation in the replication of ASFV in cells from the non-susceptible human species. Another factor contributing to the observed differential expression of ASFV proteins could be the different growth kinetics of OURT 88/3-ΔTK-GFP in the cells from different hosts (Fig. 1) which may have influenced expression profiles in the late phase. Also, proteins exclusively expressed in the early phase of infection may be underrepresented. In early studies using one-dimensional or two-dimensional electrophoresis (2DE) in combination with radioactive pulse labelling and inhibitors of viral DNA replication^21–24^ the numbers of virus induced proteins synthesized in the early and late phases of infection were assessed. Although the experimental conditions were different from ours, it is interesting to note that in the study by Esteves *et al*.^23^, 24 of the 35 early proteins and in the study by Urzainqui *et al*.^21^ all early proteins were also transcribed in the late phase. As these numbers are based on the incorporation of radioactive precursors in a pulse labelling experiment we assume that the majority of early proteins will be amenable to mass spectrometric detection also in the late phase of infection as they are still synthesized or have accumulated during infection. Indeed, a number of proteins listed in Supplementary Table S3 are expressed with early kinetics. In contrast to these early publications, MALDI-TOF MS was combined with 2DE analysis in a study focusing on the identification of host proteins modulated after ASFV infection^25^. Approximately 60% of the 68 identified protein spots were of viral origin, but the identities of these proteins and kinetics of expression were not reported.

For the qualitative analysis the use of two more proteases in addition to the most commonly used enzyme, trypsin, was beneficial for the yields and the sequence coverages of identified proteins. This observation was most striking for the comparison of tryptic and chymotryptic peptides obtained after digestion of infected WSL-HP cells. While only 31.9% of the 8,643 *Sus scrofa* specific peptides resulted from the chymotrypsin cleavage, this was the case for 44.7% of the 1,254 ASFV specific peptides. Using Fisher’s exact test, these ratios indicated a highly significant overrepresentation of chymotryptic peptides among the ASFV derived peptides. Most likely, this was a consequence of the slightly divergent physicochemical properties of ASFV proteins in comparison to the host proteins.

A prerequisite for MS based proteome analysis is the correct annotation of the ORFs within the genome sequence. We have therefore also applied a proteogenomic approach to our MS data in order to identify any additional unidentified ORFs. To this end, the genome of ASFV strain OURT 88/3 was translated in the six possible reading frames and the resulting database was used to re-evaluate the mass spectra with the Mascot search engine (data not shown). However, peptide assignments to so-far unknown potential new reading frames were not observed so that we have no indication for any additions to the annotations in the published OURT 88/3 protein sequences.

Of the 32 members of the multi gene families present in OURT 88/3, we have identified 6, specifically one MGF 505 protein (MGF 505-9 R) and five MGF 110 members (MGF 110-1L, -2L, -4L, -5L, and -14L). While MGF 110-14L and MGF 505-9 R were expressed weakly and detected only in Vero cells, the expression of the four remaining MGF 110 proteins (1L, 2L, 4L, and 5L) was stronger and, with the exception of MGF 110-2 L, they were detected in all three cell lines. While MGF 360 and MGF 530 members have been reported to be virulence determinants, interact with the innate immune system^9,26,27^, and determine the host range of ASFV^10,11^, MGF 110 genes have been shown to be non-essential for the infection of and virulence in pigs^28^. Nevertheless, MGF 110-1L, -4L and -5L are expressed in all three cell cultures suggesting they may play an at least beneficial role here. As MGF 110 proteins have been reported to contain signal peptides^29^, the coding sequences were analysed for this feature by SignalP 4.0^30^ and Phobius softwares^31,32^. Twelve predicted mature sequences were added to the sequence database for a re-evaluation of the mass spectrometric data. As shown in Supplementary Fig. S2, the N-termini of the predicted mature sequences of all four identified MFG 110 proteins 1L, 2L, 4L, and 5L and also of pI329L were confirmed by MS. For 44 additional of the 94 identified proteins the N-terminal peptides were identified (Supplementary data, Table S1). Only six were in the native amino form, while all other were modified by N-terminal acetylation, a common post-translational modification.

### Quantitative proteome analysis

**Figure 4:**
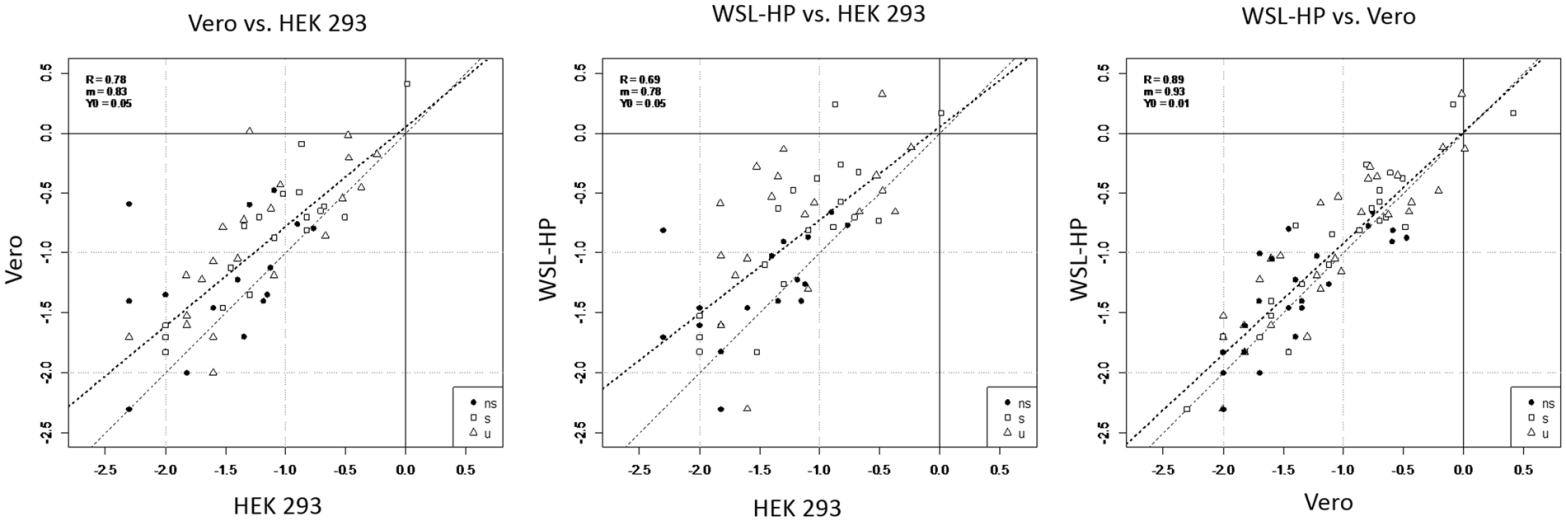
Correlation of ASFV protein expression levels in WSL-HP, HEK 293 and Vero cells. Protein abundances determined in the cells indicated at the axes are given in mole % in a logarithmic scale. Non-structural (ns) proteins are marked as full black circles, structural (s) and uncharacterized (u) proteins as open squares and triangles, respectively. Correlation coefficients are given as R, the y-intercepts and slopes of the dotted regression lines are given as Y0 and m, respectively. Dissecting lines are dashed.

**Figure 5:**
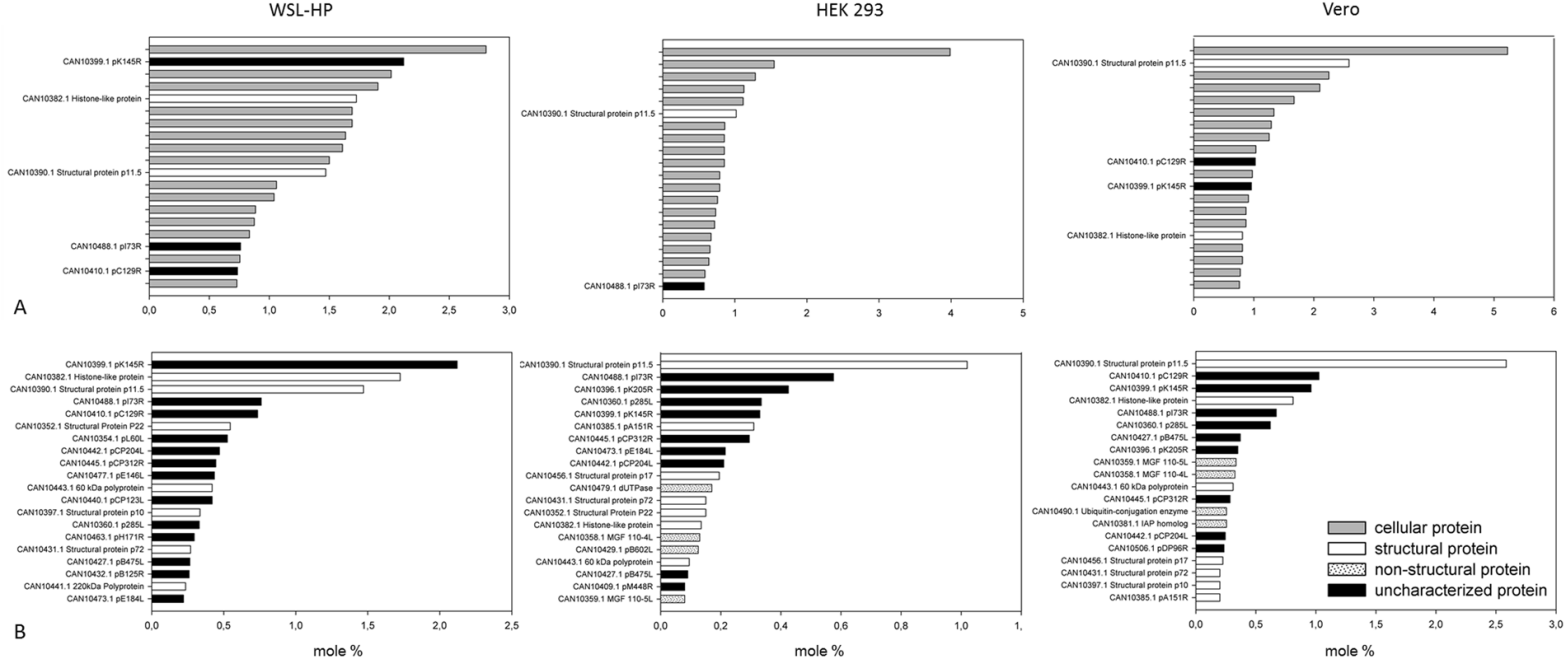
Comparison of the most abundantly expressed ASFV proteins. Protein proportions were calculated as mole percentages (x-axes) on basis of the tryptic digests using the emPAI algorithm. Panel A shows the 20 most abundant proteins (including cellular proteins as grey bars) and panel B the 20 most abundant ASFV proteins in decreasing order. Even in HEK 293 cells, which had the lowest overall content of ASFV proteins, two viral proteins were present among the 20 most abundant proteins. In all cell lines the structural protein p11.5 as well as the so far uncharacterized proteins pK145R and pI73R were expressed at high abundance. Note that in panel B, despite of some overlap between the top-ranking proteins in different cells, there are considerable differences in the abundances.

The quantitative evaluation of the MS analysis shown in Figs 4 and 5 was performed using the exponentially modified protein abundance index (emPAI)^33^, a label-free approach which allows the calculation of protein abundances on basis of the number of identified peptides that are annotated to a certain protein during the protein identification process. With 14 and 17 mole % (10.2 and 13.3 weight %) of total protein content the expression of ASFV proteins in Vero and WSL-HP was quite massive and significantly higher than in HEK 293 cells (6.3 mole %, 4.8 weight %). The lower number of identified ASFV specific proteins in HEK 293 cells correlated with the lower content of ASFV proteins present in this cell line and could therefore be a matter of sensitivity as poorly expressed proteins may have dropped below the detection limit. However, the quantitative evaluation of the MS data showed that some ASFV proteins were expressed in HEK 293 cells at similar or even higher levels than in WSL-HP or Vero cells (Fig. 4) indicating that expression of individual proteins differed markedly in the different cells and arguing against a general underrepresentation of ASFV proteins in HEK 293 cells.

Although the abundances of individual ASFV proteins varied in the different cell lines, a certain degree of correlation between the expression levels was observed which was most striking for the ASFV proteins expressed in Vero and in WSL-HP cells (Fig. 4, right panel) representing a susceptible (WSL-HP) and a non-susceptible (Vero) host. This result suggests that the course of infection in both cell lines is similar and both may be useful for infection studies with ASFV. This observation may also be helpful for the retrospective assessment of studies which have been performed with Vero cells in the past.

Non-structural proteins were found among the less expressed proteins in all three cell lines while the most abundant were structural proteins and, surprisingly, so far uncharacterized proteins. In Fig. 5, an overview of the 20 most abundant proteins in the cell extracts (including host proteins) is given in panel A and the abundances of the 20 highest ranking ASFV proteins (without cellular proteins) are compared in panel B. The abundance ranking within the ASFV proteins differed between in the three cell lines (Fig. 5B) which may reflect different relevance of the proteins in cells of the three differentially susceptible hosts. However, there also were notable similarities. Three so far uncharacterized proteins ranked among the top 20 abundant proteins in WSL-HP cells, namely pK145R, pC129R, and pI73R. Of these, pK145R and pC129R ranked also among the top 20 most abundant ASFV proteins in Vero cells. The third, pI73R, was also abundantly expressed in HEK 293 cells. The high expression levels of these proteins in all three tested cell lines may indicate important roles during ASFV infection and make them prime subjects of future investigations including rational vaccine design.

## Methods

### Virus and cells

HEK 293 (CCLV-RIE #197)^34^, WSL-HP (CCLV-RIE #1346)^35^, and Vero cells (CCLV-RIE #0015) were provided by the Biobank of the Friedrich-Loeffler-Institut. Cells were cultivated in Minimum Essential Medium (MEM) supplemented with fetal bovine serum (10%) and Penicillin/Streptomycin solution (1%, Biochrom, Berlin, Germany) at 37 °C in a humidified atmosphere with 2.5% CO_2_. All live-virus experiments were carried out in a biocontainment facility that fulfils the safety requirements for ASF laboratories and animal facilities laid out in Chapter VIII of Commission Decision 2003/422/EC. The GFP-expressing ASFV mutant OURT 88/3-ΔTK-GFP was used throughout this study. OURT 88/3-ΔTK-GFP was generated from strain OURT 88/3 (kindly provided by Linda Dixon, Pirbright, UK) as described for the corresponding NHV recombinant^17^. The correct integration of the late ASFV p72 promoter regulated GFP expression cassette was confirmed by direct sequencing of PCR amplicons spanning the mutagenized locus. To improve infection rates, the cells were inoculated in 6-well or 24-well plates by centrifugation (600 × g at 37 °C) during the 1 h incubation period (JH Forth, L Käbisch, R Portugal, S Blome, GM Keil, unpublished). The inoculum was then replaced by fresh medium and cells were further incubated.

For measuring growth kinetics cells were cultivated in 24-well plates and infected at a multiplicity of infection (MOI) of 2. After incubation for the times indicated supernatants were removed and cells were scraped into phosphate buffered saline (PBS, 250 µl), and stored at −80 °C. Progeny viral titers were determined on WSL-HP cells cultivated in 96-well plates (Eppendorf, # 0030 730.119) using serial tenfold dilutions and the method of Reed and Muench^36^. For quantitative RT-PCR and immunoblots, cell monolayers were washed with PBS and harvested in Trizol Reagent^TM^ (Invitrogen, #15596-026).

For the MS analyses, HEK 293, WSL-HP, and Vero cells were cultivated in 6-well cell culture dishes (Corning, #3506) to 90% confluency. After infection at a MOI of 2 (HEK 293) or 5 (Vero and WSL-HP) and further incubation for 24 h (HEK 293 and Vero cells) or 48 hours (WSL-HP cells) the complete cell layer was positive for GFP fluorescence. The cell monolayers were washed twice with PBS, scraped into lysis buffer (500 µl, 0.1 M Tris-HCl, pH 8.0, 0.1 M DTT, 2% SDS)^37^ and heated to 95 °C for 10 min. The lysate was cleared by centrifugation (14,000 × g, 5 min, RT), and aliquots containing extracts equivalent to 2 × 10^5^ cells were processed for MS.

### Quantitative RT-PCR

For RNA-extraction, cells were harvested in Trizol Reagent^TM^ (250 µl, Invitrogen). After addition of dichloromethane (50 µl), samples were centrifuged (13,000 rpm, 4 °C, 10 min), the aqueous phase was removed and RNA was extracted using the RNeasy Mini Kit (Qiagen, #74104) and RNase free DNase-Kit (Qiagen, # 79254). For removal of residual DNA, samples were treated with Turbo DNase (Life Technologies, #AM2238). Quantitative RT-PCR (RT-qPCR) targeting p30 and p72 was performed using standard protocols (JH Forth, L Käbisch, R Portugal, S Blome, GM Keil, unpublished).

### Polyacrylamide gel electrophoresis

Samples of cell lysates containing the equivalent of 1.5 × 10^4^ cells (approximately 15 µg protein) were mixed with sample buffer^38^, heated to 95 °C for 10 min, and separated by electrophoresis in a Mini Protean II apparatus (BioRad) using hand cast gradient (7–15% acrylamide) or homogenous (10% acrylamide) SDS-polyacrylamide gels^38^. Proteins were visualized by colloidal Coomassie Brilliant Blue staining^39^.

### Immunoblots

Proteins were purified using the Trizol Reagent workflow as described in the manual, separated on polyacrylamide gels (10%) and electroblotted to nitrocellulose membrane^40^. Blots were incubated with PBS containing 10% horse serum and 6% skimmed milk powder overnight at 4 °C. After incubation with appropriate dilutions of rabbit sera directed against p30 (1:20,000) or p72 (1:50,000) made up in blocking buffer (PBS containing 0.1% Tween-20 [PBS-T], 0.6% skimmed milk powder, and 1% horse serum) for 1 h at RT, the membranes were washed with PBS supplemented with 0.3% Tween-20 for 1 min and in PBS-T for 5 min and then incubated with secondary antibody (1:2,000 dilution in PBS-T, peroxidase-conjugated goat anti-rabbit, Dianova, #111-036-045) for 1 h at RT. Membranes were washed as above, and bound antibodies were detected using the Clarity Western ECL substrate (BioRad, # 170-5061).

### Mass spectrometric workflow

Proteome analysis was performed using a shotgun approach. Samples were digested into peptides, which were separated by nano liquid reversed phase chromatography (EASY-nLC II, Bruker), and spotted to a MALDI target by a Proteineer fcII sample spotting robot (Bruker). Mass spectrometric analysis was performed on an UltrafleXtreme MALDI-TOF/TOF instrument (Bruker). All reagents used were of highest purity available, all solvents were of MS grade.

### Protein digest

Proteins were digested by filter-aided sample preparation (FASP)^41^ using a Vivacon 500 filter unit (MWCO 30 kDa, Sartorius, #VN01H22) with slight adjustments of the buffer compositions for the three different proteases. Trypsin (Promega, #V5111) digest was carried out in NH_4_HCO_3_ buffer (0.05 M, pH 7.8), while endoproteinase Glu-C (V8 protease from *Staphylococcus aureus*, Promega, #V165A) and chymotrypsin (Promega, #V106A) digests were performed in Tris(hydroxymethyl)-aminomethane (Tris-HCl) buffer (0.1 M, pH 7.5). Digest was carried out for 16 h at 37 °C (trypsin and Glu-C) or 25 °C (chymotrypsin) with enzyme:substrate ratios of 1:50. After filtration peptides were desalted with Ziptip C18 solid phase extraction tips (Merck Millipore, # ZTC18S960), dried under vacuum and dissolved in trifluoroacetic acid (TFA) solution (0.1% v/v) to a concentration of 2 μg/μl prior to nano-LC.

### Nano reversed phase liquid chromatography of peptides

Peptide samples equivalent to 10 µg protein^42^ were diluted in TFA (0.1%) and applied onto a NS-MP-10 loading/desalting column (C18-modified silica gel, 5 µm bead size, inner diameter 100 µm, length 20 mm, BioSphere). After washing with TFA (40 µl, 0.05%, 5 µl/min) peptides were eluted onto the analytical column (Acclaim PepMap100, 75 µm × 15 cm, C18, 3 µm, 100 Å; Thermo Scientific, #160321) at a flow rate of 300 nl/min by application of an acetonitrile (ACN) gradient with the following profile (solvent A: 0.05% TFA, solvent B: 90% ACN, 0.05% TFA): 2% to 9% solvent B (0–15 min), 9% to 32% solvent B (15–211 min), 32% to 45% solvent B (211–226 min) and 100% solvent B (226–238 min). Fractions were collected every 10 s. Eluted peptides were mixed with an α-cyano-hydroxycinnamic acid matrix solution (0.416 µl per fraction) made up as suggested by the manufacturer (Bruker).

### MALDI-TOF/TOF mass spectrometry

Mass spectrometric analysis was performed in positive mode in the m/z range from 700 to 3,500 Da. A maximum of 40 peptide peaks per fraction with signal-to-noise ratios above 5 were selected for fragmentation. The spectra were processed with Flexanalysis software (version 3.4, Bruker) and the proteins were identified by the Mascot search engine (version 2.4.1, Matrix Science) using the following settings: peptide and fragment mass tolerance were set to 25 ppm and 0.7 Da, respectively. Oxidation of methionine and the acetylation of protein N-termini were set as variable modifications, whereas the carbamidomethylation of cysteine was set as fixed modification. One missed cleavage site was tolerated for tryptic digests, up to 6 for samples digested with Glu-C or chymotrypsin. The false discovery rate was set to a maximum of 2%. For the Mascot search of samples from the different species databases representing the human and the domestic pig proteome were downloaded from the Ensembl repository^43^ while the *Chlorocebus sabaeus* proteome was downloaded from the Uniprot Knowledgebase^44^. Proteins specified by the used ASFV strain OURT 88/3 (GenBank AM712240.1^45^), were added to all three host cell proteomes and the appropriate compilation of viral and host cell proteome was used for the different samples. The results of the Mascot database search were exported to the ProteinScape software (Version 3.1, Bruker). Only proteins identified with at least two peptides exceeding the Mascot peptide identification score are reported. Proteins were quantified using the exponentially modified protein abundance index (emPAI)^46,47^ on basis of the MS results of the tryptic digests.

### Data evaluation

The following software was used for the construction of figures and Tables: CFX Manager (version. 3.1, BioRad), Aida Image Analyser (version 5.0, Raytest), Excel 2016 (Microsoft), SigmaPlot 11 (version 11, Systat) and the statistical programming language R^48^.

## Electronic supplementary material

Supplementary Information

Supplementary MS Data
