# Supplementary material for: The intracellular proteome of African swine fever virus
Source: Sci Rep. 2018 Oct 2;8:14714. doi: 10.1038/s41598-018-32985-z (PMC6168524; doi:10.1038/s41598-018-32985-z)

The intracellular proteome of African swine fever virus

Catharina Keßler[1], Jan Hendrik Forth[2], Günther M. Keil[1], Thomas C. Mettenleiter[1], Sandra Blome[3], Axel Karger[1*]

[1] Institute of Molecular Virology and Cell Biology

[2] Institute of Infectology

[3] Institute of Diagnostic Virology

Friedrich-Loeffler-Institut, Federal Research Institute for Animal Health, Südufer 10, 17493 Greifswald-Insel Riems, Germany

[*]Corresponding author:
Dr. Axel Karger, Institute of Molecular Virology and Cell Biology,
Friedrich-Loeffler-Institut
Federal Research Institute of Animal Health
Südufer 10
D-17493 Greifswald-Insel Riems/ Germany
Phone: +49 383517-1247
e-mail: axel.karger@fli.de

Supplementary Table S3. Summary of the MS results obtained for ASFV-specific proteins. The number of identified peptides (Pep), the sequence coverages (SC), and Mascot protein identification scores (Score) are given for every identified ASFV protein and for every cell line. Column 'Class' indicates structural (s), non-structural (ns), and so far uncharacterised (u) proteins and the current knowledge about the expression of the respective gene (O, predicted ORF; P, evidence for protein; R, evidence for mRNA). Proteins with an annotated function in column 'Description' which lack evidence for incorporation into the virions are listed as non-structural (ns). Data in this column was compiled from the Uniprot knowledgebase [1], review articles [2,3,4], and individual references given in column 'Ref'.

Accession	Gene	Description	WSL-HP			HEK 293			Vero			Class	Ref
			Pep	SC	Score	Pep	SC	Score	Pep	SC	Score		
**CAN10352.1**	KP177R	Structural protein P22	22	71.2	1756	13	56.5	592	17	69.5	1095	s P	5,6
**CAN10354.1**	L60L	pL60L	10	95.5	987	3	85.1	193	10	100	837	u R	7
**CAN10356.1**	MGF 110-1L	MGF 110-1L	6	23.3	324	11	31.1	465	16	48.5	778	ns R	8
**CAN10357.1**	MGF 110-2L	MGF 110-2L	5	52.9	346	0	0	0	2	17.3	67	ns R	8
**CAN10358.1**	MGF 110-4L	XP124L. Multigene family 110 member	18	73.4	946	14	60.5	706	26	62.9	1452	s P	8,9
**CAN10359.1**	MGF 110-5L	MGF 110-5L	15	55.6	670	11	46	479	28	60.5	1398	ns R	8
**CAN10360.1**	285L	p285L	9	70.2	524	10	71.3	455	11	71.3	586	u O	
**CAN10365.1**	MGF 110-14L	MGF 110-14L	0	0	0	0	0	0	3	28.6	124	ns R	8
**CAN10379.1**	MGF 505-9R	MGF 505-9R	0	0	0	0	0	0	2	3.8	90	ns R	10,11
**CAN10381.1**	A224L	AP apoptosis inhibitor	15	67.4	624	0	0	0	18	66.1	755	ns P	12
**CAN10382.1**	A104R	Histone-like	22	90.4	1738	14	90.4	801	17	90.4	1092	s P	13-15
**CAN10383.1**	A240L	Thymidylate kinase	2	10	68	0	0	0	0	0	0	ns R	16
**CAN10385.1**	A151R	pA151R	12	71.5	746	13	64.2	598	14	68.9	673	s P	17
**CAN10390.1**	A137R	p11.5	44	98.5	3104	40	99.3	2338	42	99.3	2498	s P	18
**CAN10391.1**	F317L	pF317L	15	38.2	565	5	19.6	147	10	36.3	381	u O	
**CAN10392.1**	F334L	Ribonucleotide reductase(small subunit)	16	39.2	727	15	41.6	712	12	30.8	603	ns O	19
**CAN10393.1**	F778R	Ribonucleotide reductase (large subunit)	18	27.4	723	15	25.7	572	13	20.6	600	ns O	19
**CAN10394.1**	F165R	pF165R	12	47.3	600	2	15.2	131	7	41.2	456	u O	
**CAN10396.1**	K205R	pK205R	39	83.4	1959	40	84.9	1997	39	83.4	1842	u P	20
**CAN10397.1**	K78R	p10	10	60.3	557	6	59	192	10	60.3	581	s P	21,22
**CAN10399.1**	K145R	pK145R	35	90.3	2174	23	78.6	1220	31	89	1706	u P	23
**CAN10400.1**	K421R	pK421R	6	14	174	0	0	0	0	0	0	u R	
**CAN10401.1**	EP1242L	RNA polymerase subunit 2	18	19.3	748	11	12.1	418	22	24.2	713	s P	24

Accession	Gene	Description	WSL-HP			HEK 293			Vero			Class	Ref
			Pep	SC	Score	Pep	SC	Score	Pep	SC	Score		
**CAN10402.1**	EP84R	pEP84R	3	19	164	0	0	0	3	19	123	ns R	
**CAN10403.1**	EP424R	FTS J-like methyl transferase domain	4	17.5	218	3	11.3	71	0	0	0	ns R	[7]
**CAN10408.1**	M1249L	PM1249L	28	29.1	898	6	4.9	236	14	15.1	472	u R	[7]
**CAN10409.1**	M448R	PM448R	22	42.6	1391	14	39.3	764	17	41.7	959	u R	[7]
**CAN10410.1**	C129R	PC129R	20	94.6	1192	2	19.4	141	21	90.7	1011	u R	[7]
**CAN10412.1**	C717R	pC717R	2	4.2	98	0	0	0	0	0	0	u R	[7]
**CAN10413.1**	C122R	pC122R	0	0	0	0	0	0	3	41	101	u O	
**CAN10414.1**	C257L	PC257L	11	37	538	0	0	0	8	37.4	420	u O	
**CAN10415.1**	C475L	PolyA polymerase large subunit	13	27.4	432	0	0	0	5	10.7	146	s O	
**CAN10416.1**	C315R	TFIIB like	5	20	274	0	0	0	3	11.4	161	ns O	
**CAN10417.1**	C147L	RNA polymerase subunit 6	0	0	0	2	13.6	81	2	13.6	82	s P	[25]
**CAN10420.1**	C962R	DNA primase	17	21.9	536	5	5.8	205	25	28.5	920	ns R	[26]
**CAN10422.1**	B962L	Helicase superfamily II	4	5.5	124	0	0	0	4	4.9	116	ns R	[19]
**CAN10423.1**	B119L	ERV 1-like/ Sulfhydryl oxidase pb119l	9	47.9	450	0	0	0	7	31.1	187	s P	[17,27]
**CAN10424.1**	B318L	Prenyltransferase	4	17.9	124	0	0	0	0	0	0	ns P	[28,29]
**CAN10425.1**	B438L	p49	8	23.5	309	0	0	0	8	21.2	211	s P	[30]
**CAN10427.1**	B475L	pB475L	52	75.2	3237	20	39.8	783	63	82.1	3597	u O	
**CAN10428.1**	B354L	pB354L	5	14.1	144	0	0	0	0	0	0	u O	
**CAN10429.1**	B602L	Chaperone	60	61.4	3530	42	52.5	1925	59	55	3328	ns P	[31]
**CAN10431.1**	B646L	p72	108	82.5	8084	42	61	2160	74	71.1	4532	s P	[32,33]
**CAN10432.1**	B125R	pB125R	15	69.6	627	3	35.2	135	6	50.4	251	u O	
**CAN10434.1**	B407L	pB407L	25	54.4	1380	8	21.1	383	11	25.5	543	u R	[7]
**CAN10435.1**	B175L	VV VLTF2-like	6	25.7	204	0	0	0	5	23.4	157	ns R	[7]
**CAN10436.1**	B263R	pB263R	0	0	0	0	0	0	2	9.5	79	u R	[7]
**CAN10438.1**	G1340L	VV A8L-like transcription factor	8	6.8	280	0	0	0	5	3.9	180	ns R	[7]
**CAN10439.1**	G1211R	DNA polymerase family B	6	7	318	11	10.7	360	13	14	398	ns R	[34,35]
**CAN10440.1**	CP123L	pCP123L	21	59.3	1306	2	22	125	13	56.9	557	u O	
**CAN10441.1**	CP2475L	pp220	252	71.6	13744	68	29.4	2702	176	58.1	9029	s P	[36-39]
**CAN10442.1**	CP204L	p32 (p30)	27	65.5	2196	23	65.5	1452	22	67.5	1456	s P	[40,41]
**CAN10443.1**	CP530R	pp62 (pp60)	64	77	4278	24	53.2	1031	58	71.7	2982	s P	[36,39]
**CAN10444.1**	CP80R	RNA polymerase subunit 10	4	43.8	141	0	0	0	4	35	129	s P	
**CAN10445.1**	CP312R	pCP312R	30	61.5	2095	28	73.1	1670	26	61.9	1569	u P	[23,42]
**CAN10447.1**	O61R	p12	2	26.2	160	0	0	0	0	0	0	s P	[39,43,44]
**CAN10448.1**	NP1450L	RNA polymerase subunit 1	14	10.7	544	9	6.6	352	13	11.6	468	s P	[24]
**CAN10449.1**	NP419L	DNA ligase	12	28.2	427	10	24.1	418	11	24.6	333	ns R	[45]
**CAN10450.1**	NP868R	Guanylyl transferase	6	7.6	309	2	2	122	4	6.2	143	s P	[46]
**CAN10451.1**	D250R	Nudix hydrolase	5	20.8	121	0	0	0	6	23.2	175	ns R	[47-50]

Accession	Gene	Description	WSL-HP			HEK 293			Vero			Class	Ref
			Pep	SC	Score	Pep	SC	Score	Pep	SC	Score		
**CAN10453.1**	D79L	pD79L	3	55.7	123	0	0	0	4	57	221	u O	26
**CAN10454.1**	D339L	pD339L	9	15.6	217	6	17.4	217	9	28	339	u O	48
**CAN10455.1**	D1133L	Helicase superfamily II	12	13.1	405	2	2.3	129	11	12.9	394	ns P	24
**CAN10456.1**	D117L	P17	11	58.1	860	9	54.7	486	13	64.1	651	s P	48,51,52
**CAN10457.1**	D205R	RNA polymerase subunit 5	0	0	0	3	17.6	100	2	11.2	117	s P	48
**CAN10458.1**	D345L	Lambda-like exonuclease	7	21.7	262	0	0	0	7	17.4	150	ns R	53
**CAN10459.1**	S183L	pS183L	3	19.1	137	0	0	0	0	0	0	u R	53
**CAN10460.1**	S273R	SUMO-1-like protease.	6	33	231	0	0	0	6	32.2	181	s P	54
**CAN10461.1**	P1192R	DNA topoisomerase type II	4	4.4	153	0	0	0	9	10.7	298	ns R	54
**CAN10462.1**	H359L	RNA polymerase subunit 3	14	44.6	716	8	31.5	358	12	35.1	567	s P	48,55
**CAN10463.1**	H171R	pH171R	22	86	1407	10	67.8	555	16	71.3	983	u R	48
**CAN10464.1**	H124R	pH124R	2	19.4	57	0	0	0	2	19.4	45	u R	7
**CAN10465.1**	H339R	pH339R (j4R)	27	58.7	1173	9	22.1	289	26	57.8	992	ns P	48
**CAN10467.1**	H233R	pH233R	6	27.5	227	0	0	0	3	13.3	96	u O	48
**CAN10468.1**	H240R	pH240R	7	22.1	252	0	0	0	4	20.8	126	u O	48
**CAN10469.1**	R298L	Serine protein kinase	4	16.8	95	0	0	0	3	12.8	75	s P	56
**CAN10470.1**	Q706L	Helicase superfamily II	6	11.9	221	4	7.9	162	10	16.6	428	ns O	57
**CAN10473.1**	E184L	pE184L	31	77.7	1477	20	62	880	22	72.3	1000	u O	23,48
**CAN10474.1**	E183L	p54 (j13L)	10	18	533	6	16.4	296	10	25.7	457	s P	58,59
**CAN10477.1**	E146L	pE146L	14	65.8	849	4	39.7	196	10	66.4	579	u P	48,60
**CAN10479.1**	E165R	dUTPase	14	68.5	677	7	53.3	442	11	66.7	558	ns O	48
**CAN10480.1**	E248R	E248R (k2R)	2	15.3	214	0	0	0	0	0	0	s P	48,61
**CAN10481.1**	E120R	p14.5	10	62.5	897	6	43.3	260	7	42.5	535	s P	62,63
**CAN10482.1**	E296R	AP endonuclease class II	9	33.1	489	10	33.4	415	5	19.9	221	ns P	48
**CAN10485.1**	I267L	pI267L	6	23.2	186	3	9.7	77	6	23.2	236	u O	48
**CAN10486.1**	I226R	pI226R	9	32.3	322	0	0	0	8	27	240	u R	48
**CAN10487.1**	I243L	Transcription factor SII	13	42.8	658	6	24.3	253	8	30.5	383	ns R	64
**CAN10488.1**	I73R	pI73R	15	97.3	1251	12	100	760	14	100	952	u R	64
**CAN10489.1**	I329L	pI329L	23	49.5	1382	0	0	0	7	22.5	249	ns R	48,64-66
**CAN10490.1**	I215L	Ubiquitin conjugating enzyme	12	40.5	532	10	25.1	387	25	76.3	1200	ns P	64,67,68
**CAN10491.1**	I177L	pI177L	2	17.5	54	0	0	0	0	0	0	u R	64
**CAN10492.1**	I196L	pI196L	2	10.2	78	0	0	0	0	0	0	u R	64
**CAN10493.1**	DP238L	pDP238L	10	34.5	404	8	22.3	289	13	42.9	629	u O	
**CAN10506.1**	DP96R	pDP96R	6	66.7	300	3	32.3	94	8	74	418	u O	

Supplementary Figure S1. Physicochemical properties of ASFV and Sus scrofa proteins. The three panels show the distributions of molecular weights (left), isoelectric points (central) and grand averages of hydropathy (GRAVY, right) of ASFV (triangles) and porcine (circles) proteins. The y-axes represent the normalized counts of proteins. Note that ASFV proteins (triangles) are, on average, smaller, more basic, and more hydrophobic than porcine proteins (circles).

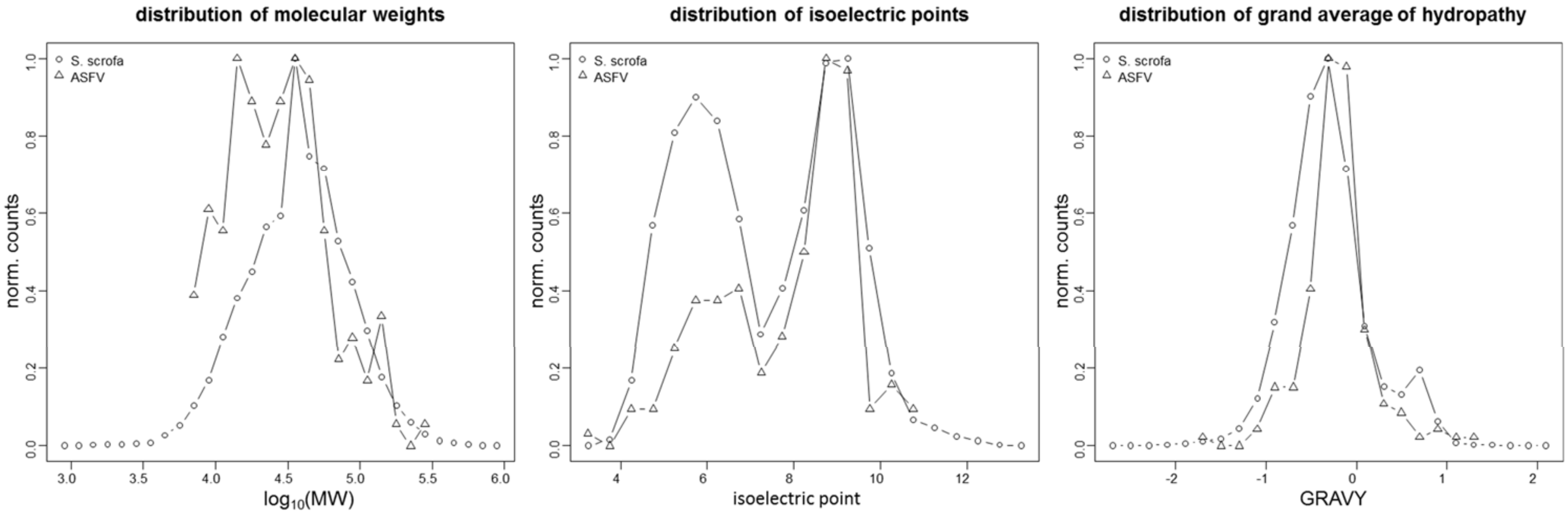

Supplementary Figure S2. Processing of signal peptides of four MGF 110 proteins and pI329L. Panel headers A-E indicate the protein name, the predicted sequence region after cleavage of the protein N-terminal signal peptide, and the sequence of the identified N-terminal peptide with a dot at the proteolytic cleavage site. Identified peptides are mapped to the mature protein sequence as grey boxes, the N-terminal peptides are highlighted as green boxes. Small red boxes indicate amino acid residues identified by peptide fragmentation. Below the protein sequences the fragment spectra of the N-terminal peptides are shown together with the annotated peptide fragment series.

A. MGF 110-1L, AA 19-270, NH$_2$-YEIEPLER.T

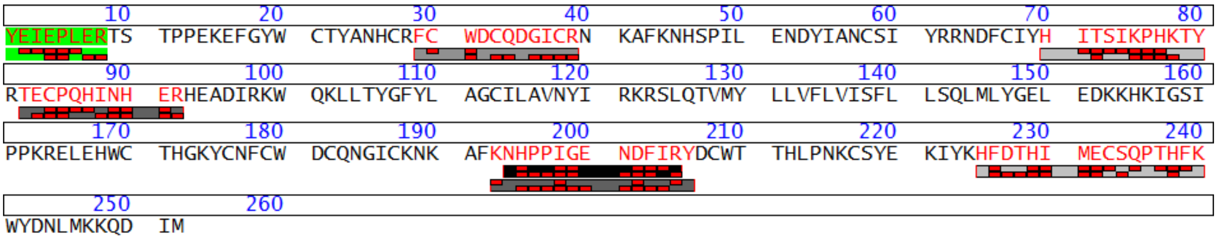

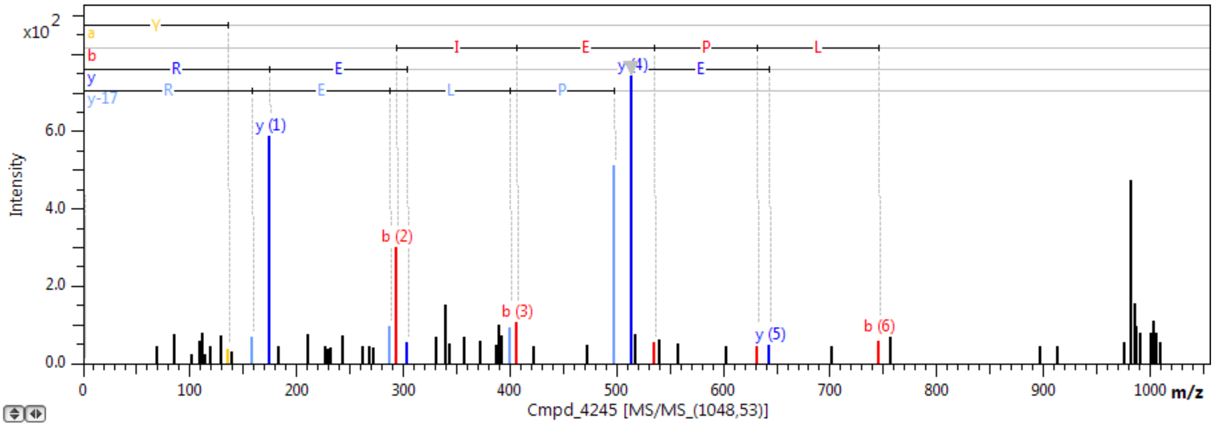

B. MGF 110-2L, AA 20-104, NH_2-FSTDNLLEEELR.Y

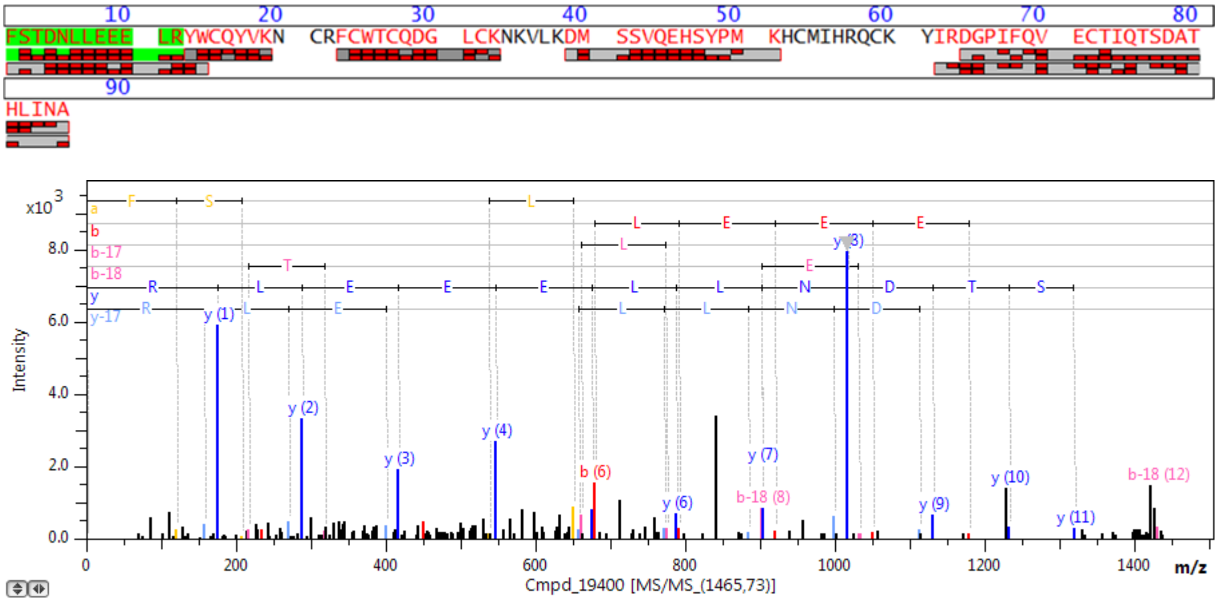

C. MGF 110-4L, AA 19-124, NH_2.LPTQAEGHLR.S

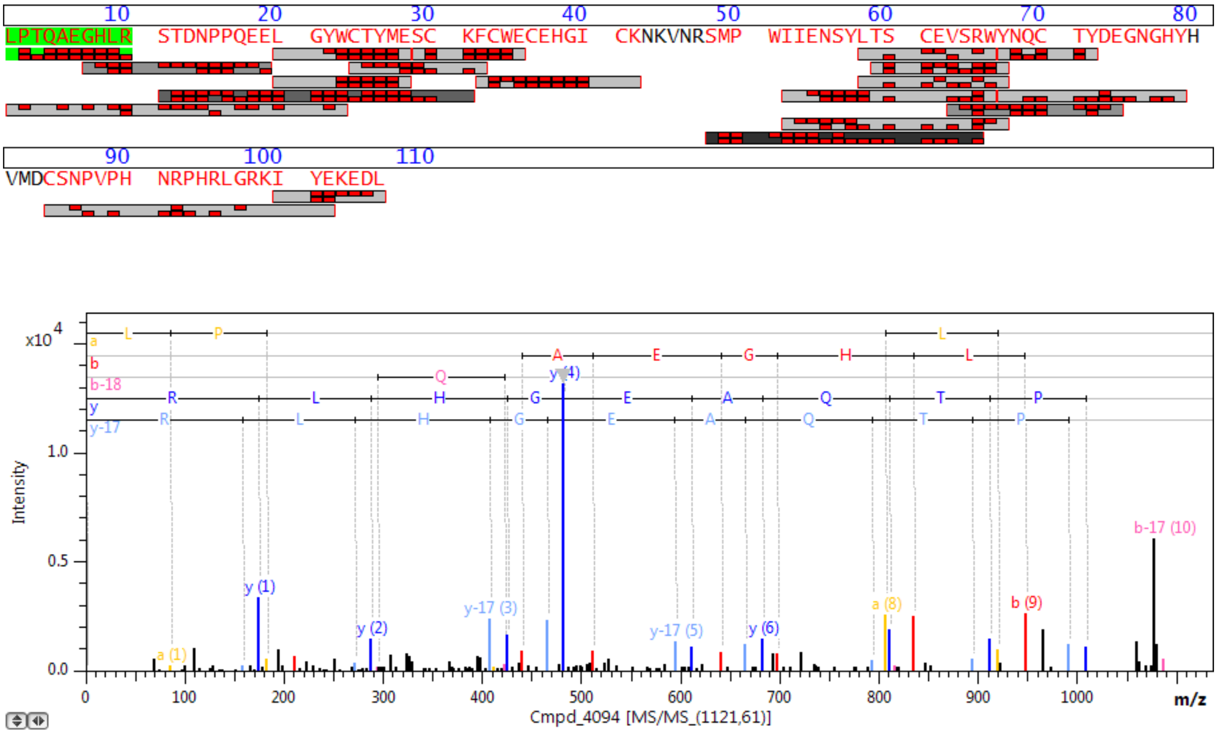

D. MGF 110-5L, AA 19-124, NH2-LPTQAGGHLR.S

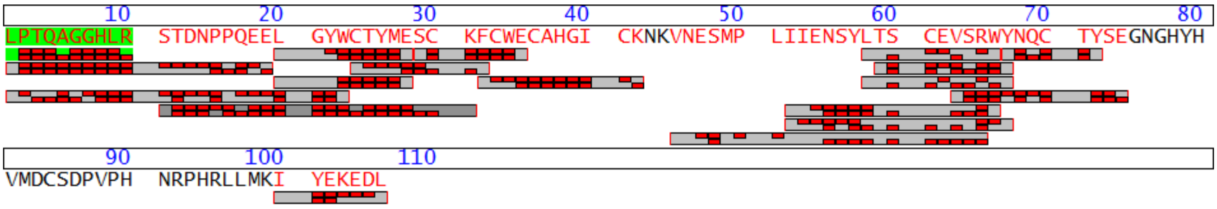

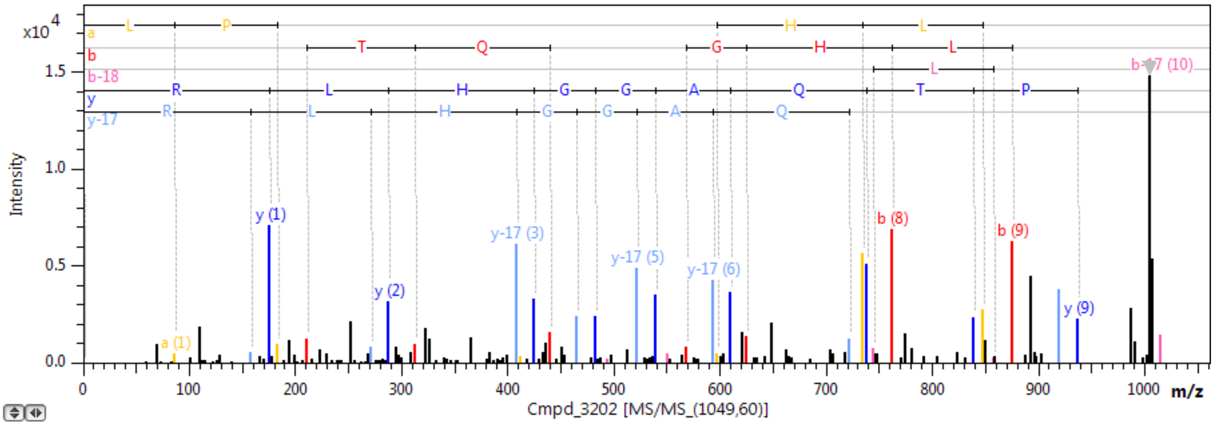

E. pI329L, AA 17-329, NH2-GRIKPQVTCKYFISE.N

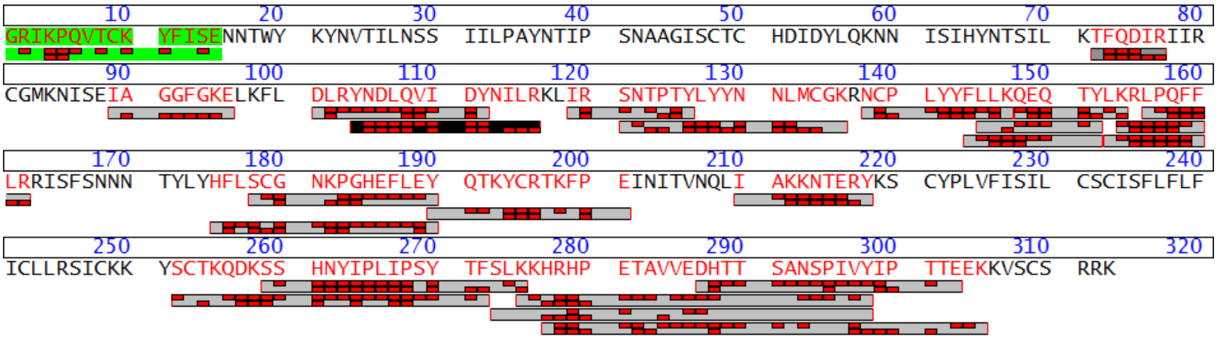

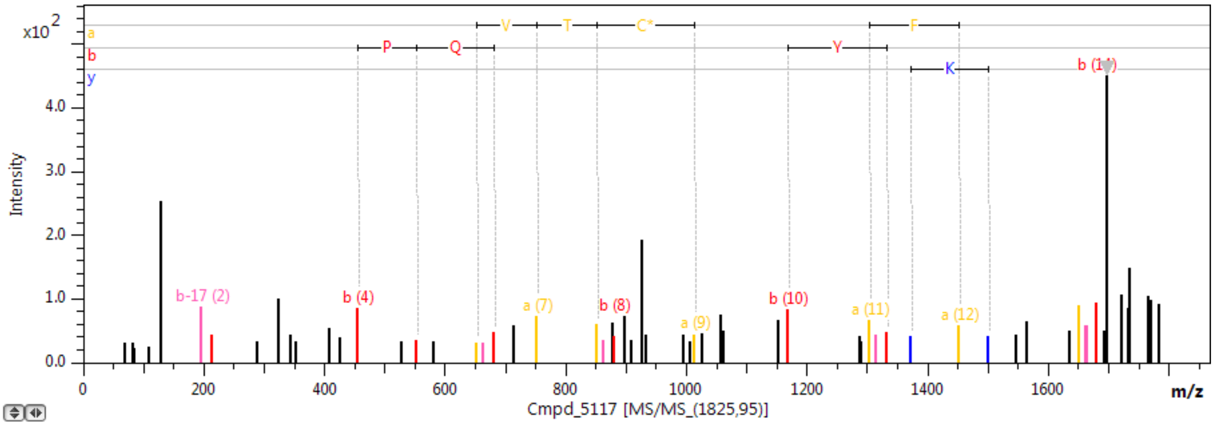

References

1 The UniProt Consortium. UniProt: the universal protein knowledgebase. *Nucleic Acids Res* **45**, D158-D169, doi:10.1093/nar/gkw1099 (2017).

2 Dixon, L. K., Chapman, D. A., Netherton, C. L. & Upton, C. African swine fever virus replication and genomics. *Virus Res* **173**, 3-14, doi:10.1016/j.virusres.2012.10.020 (2013).

3 Dixon, L. K., Alonso, C., Escribano, J.M., Martins, C., Revilla, Y., Salas, M.L. and Takamatsu, H. in *Virus Taxonomy Classification and Nomenclature of Viruses Ninth Report of the International Committee on Taxonomy of Viruses Virus Taxonomy Classification and Nomenclature of Viruses Ninth Report of the International Committee on Taxonomy of Viruses* (ed Michael J. Adams Andrew M.Q. King, Eric B. Carstens, and Elliot J. Lefkowitz) 153-162 (Elsevier Academic Press, 2012).

4 Yanez, R. J. *et al.* Analysis of the complete nucleotide sequence of African swine fever virus. *Virology* **208**, 249-278, doi:10.1006/viro.1995.1149 (1995).

5 Camacho, A. & Vinuela, E. Protein p22 of African swine fever virus: an early structural protein that is incorporated into the membrane of infected cells. *Virology* **181**, 251-257 (1991).

6 Vydelingum, S., Baylis, S. A., Bristow, C., Smith, G. L. & Dixon, L. K. Duplicated genes within the variable right end of the genome of a pathogenic isolate of African swine fever virus. *J Gen Virol* **74 (Pt 10)**, 2125-2130 (1993).

7 Jaing, C. *et al.* Gene expression analysis of whole blood RNA from pigs infected with low and high pathogenic African swine fever viruses. *Sci Rep* **7**, 10115, doi:10.1038/s41598-017-10186-4 (2017).

8 Almendral, J. M., Almazan, F., Blasco, R. & Vinuela, E. Multigene families in African swine fever virus: family 110. *J Virol* **64**, 2064-2072 (1990).

9 Rouiller, I., Brookes, S. M., Hyatt, A. D., Windsor, M. & Wileman, T. African swine fever virus is wrapped by the endoplasmic reticulum. *J Virol* **72**, 2373-2387 (1998).

10 Rodriguez, J. M. *et al.* Multigene families in African swine fever virus: family 505. *J Virol* **68**, 2746-2751 (1994).

11 Yozawa, T., Kutish, G. F., Afonso, C. L., Lu, Z. & Rock, D. L. Two novel multigene families, 530 and 300, in the terminal variable regions of African swine fever virus genome. *Virology* **202**, 997-1002, doi:10.1006/viro.1994.1426 (1994).

12 Chacon, M. R., Almazan, F., Nogal, M. L., Vinuela, E. & Rodriguez, J. F. The African swine fever virus IAP homolog is a late structural polypeptide. *Virology* **214**, 670-674, doi:10.1006/viro.1995.0083 (1995).

13 Borca, M. V. *et al.* African swine fever virus structural protein p72 contains a conformational neutralizing epitope. *Virology* **201**, 413-418, doi:10.1006/viro.1994.1311 (1994).

14 Borca, M. V. *et al.* A structural DNA binding protein of African swine fever virus with similarity to bacterial histone-like proteins. *Arch Virol* **141**, 301-313 (1996).

15 Frouco, G. *et al.* DNA-binding properties of the African swine fever virus pA104R, a histone-like protein involved in viral replication and transcription. *J Virol*, doi:10.1128/jvi.02498-16 (2017).

16 Yanez, R. J., Rodriguez, J. M., Rodriguez, J. F., Salas, M. L. & Vinuela, E. African swine fever virus thymidylate kinase gene: sequence and transcriptional mapping. *J Gen Virol* **74 (Pt 8)**, 1633-1638 (1993).

17 Rodriguez, I. *et al.* African swine fever virus pB119L protein is a flavin adenine dinucleotide-linked sulfhydryl oxidase. *J Virol* **80**, 3157-3166, doi:10.1128/JVI.80.7.3157-3166.2006 (2006).

18 Alcami, A., Angulo, A. & Vinuela, E. Mapping and sequence of the gene encoding the African swine fever virion protein of M(r) 11500. *J Gen Virol* **74 (Pt 11)**, 2317-2324, doi:10.1099/0022-1317-74-11-2317 (1993).

19 Boursnell, M., Shaw, K., Yanez, R. J., Vinuela, E. & Dixon, L. The sequences of the ribonucleotide reductase genes from African swine fever virus show considerable homology with those of the orthopoxvirus, vaccinia virus. *Virology* **184**, 411-416 (1991).

20 Gutierrez-Castaneda, B., Reis, A. L., Corteyn, A., Parkhouse, R. M. & Kollnberger, S. Expression, cellular localization and antibody responses of the African swine fever virus genes B602L and K205R. *Arch Virol* **153**, 2303-2306, doi:10.1007/s00705-008-0246-z (2008).

21 Salas, M. L., Salas, J. & Vinuela, E. Phosphorylation of African swine fever virus proteins in vitro and in vivo. *Biochimie* **70**, 627-635 (1988).

22 Munoz, M., Freije, J. M., Salas, M. L., Vinuela, E. & Lopez-Otin, C. Structure and expression in E. coli of the gene coding for protein p10 of African swine fever virus. *Arch Virol* **130**, 93-107 (1993).

23 Kollnberger, S. D., Gutierrez-Castaneda, B., Foster-Cuevas, M., Corteyn, A. & Parkhouse, R. M. Identification of the principal serological immunodeterminants of African swine fever virus by screening a virus cDNA library with antibody. *J Gen Virol* **83**, 1331-1342, doi:10.1099/0022-1317-83-6-1331 (2002).

24 Yanez, R. J., Boursnell, M., Nogal, M. L., Yuste, L. & Vinuela, E. African swine fever virus encodes two genes which share significant homology with the two largest subunits of DNA-dependent RNA polymerases. *Nucleic Acids Res* **21**, 2423-2427 (1993).

25 Lu, Z., Kutish, G. F., Sussman, M. D. & Rock, D. L. An African swine fever virus gene with a similarity to eukaryotic RNA polymerase subunit 6. *Nucleic Acids Res* **21**, 2940 (1993).

26 Yanez, R. J., Rodriguez, J. M., Boursnell, M., Rodriguez, J. F. & Vinuela, E. Two putative African swine fever virus helicases similar to yeast 'DEAH' pre-mRNA processing proteins and vaccinia virus ATPases D11L and D6R. *Gene* **134**, 161-174 (1993).

27 O'Donnell, V. *et al.* African Swine Fever Virus Georgia 2007 with a Deletion of Virulence-Associated Gene 9GL (B119L), when Administered at Low Doses, Leads to Virus Attenuation in Swine and Induces an Effective Protection against Homologous Challenge. *J Virol* **89**, 8556-8566, doi:10.1128/jvi.00969-15 (2015).

28 Alejo, A., Andres, G., Vinuela, E. & Salas, M. L. The African swine fever virus prenyltransferase is an integral membrane trans-geranylgeranyl-diphosphate synthase. *J Biol Chem* **274**, 18033-18039 (1999).

29 Alejo, A., Yanez, R. J., Rodriguez, J. M., Vinuela, E. & Salas, M. L. African swine fever virus trans-prenyltransferase. *J Biol Chem* **272**, 9417-9423 (1997).

30 Epifano, C., Krijnse-Locker, J., Salas, M. L., Rodriguez, J. M. & Salas, J. The African swine fever virus nonstructural protein pB602L is required for formation of the icosahedral capsid of the virus particle. *J Virol* **80**, 12260-12270, doi:10.1128/JVI.01323-06 (2006).

31 Cobbold, C., Windsor, M. & Wileman, T. A virally encoded chaperone specialized for folding of the major capsid protein of African swine fever virus. *J Virol* **75**, 7221-7229, doi:10.1128/jvi.75.16.7221-7229.2001 (2001).

32 Cistue, C. & Tabares, E. Expression in vivo and in vitro of the major structural protein (VP73) of African swine fever virus. *Arch Virol* **123**, 111-124 (1992).

33 Garcia-Escudero, R., Andres, G., Almazan, F. & Vinuela, E. Inducible gene expression from African swine fever virus recombinants: analysis of the major capsid protein p72. *J Virol* **72**, 3185-3195 (1998).

34 Rodriguez, J. M., Yanez, R. J., Rodriguez, J. F., Vinuela, E. & Salas, M. L. The DNA polymerase-encoding gene of African swine fever virus: sequence and transcriptional analysis. *Gene* **136**, 103-110 (1993).

35 Martins, A., Ribeiro, G., Marques, M. I. & Costa, J. V. Genetic identification and nucleotide sequence of the DNA polymerase gene of African swine fever virus. *Nucleic Acids Res* **22**, 208-213 (1994).

36 Simon-Mateo, C., Andres, G., Almazan, F. & Vinuela, E. Proteolytic processing in African swine fever virus: evidence for a new structural polyprotein, pp62. *J Virol* **71**, 5799-5804 (1997).

37 Simon-Mateo, C., Andres, G. & Vinuela, E. Polyprotein processing in African swine fever virus: a novel gene expression strategy for a DNA virus. *EMBO J* **12**, 2977-2987 (1993).

38 Andres, G., Simon-Mateo, C. & Vinuela, E. Assembly of African swine fever virus: role of polyprotein pp220. *J Virol* **71**, 2331-2341 (1997).

39 Andres, G., Alejo, A., Salas, J. & Salas, M. L. African swine fever virus polyproteins pp220 and pp62 assemble into the core shell. *J Virol* **76**, 12473-12482 (2002).

40 Afonso, C. L. *et al.* Characterization of p30, a highly antigenic membrane and secreted protein of African swine fever virus. *Virology* **189**, 368-373 (1992).

41 Prados, F. J., Vinuela, E. & Alcami, A. Sequence and characterization of the major early phosphoprotein p32 of African swine fever virus. *J Virol* **67**, 2475-2485 (1993).

42 Reis, A. L., Parkhouse, R. M., Penedos, A. R., Martins, C. & Leitao, A. Systematic analysis of longitudinal serological responses of pigs infected experimentally with African swine fever virus. *J Gen Virol* **88**, 2426-2434, doi:10.1099/vir.0.82857-0 (2007).

43 Carrascosa, A. L., Saastre, I., Gonzalez, P. & Vinuela, E. Localization of the African swine fever virus attachment protein P12 in the virus particle by immunoelectron microscopy. *Virology* **193**, 460-465, doi:10.1006/viro.1993.1146 (1993).

44 Salas, M. L. & Andres, G. African swine fever virus morphogenesis. *Virus Res* **173**, 29-41, doi:10.1016/j.virusres.2012.09.016 (2013).

45 Yanez, R. J. & Vinuela, E. African swine fever virus encodes a DNA ligase. *Virology* **193**, 531-536, doi:10.1006/viro.1993.1161 (1993).

46 Pena, L., Yanez, R. J., Revilla, Y., Vinuela, E. & Salas, M. L. African swine fever virus guanylyltransferase. *Virology* **193**, 319-328, doi:10.1006/viro.1993.1128 (1993).

47 Koonin, E. V. & Senkevich, T. G. Vaccinia virus encodes four putative DNA and/or RNA helicases distantly related to each other. *J Gen Virol* **73 (Pt 4)**, 989-993, doi:10.1099/0022-1317-73-4-989 (1992).

48 Dixon, L. K. *et al.* Nucleotide sequence of a 55 kbp region from the right end of the genome of a pathogenic African swine fever virus isolate (Malawi LIL20/1). *J Gen Virol* **75 (Pt 7)**, 1655-1684, doi:10.1099/0022-1317-75-7-1655 (1994).

49 Cartwright, J. L. *et al.* The g5R (D250) gene of African swine fever virus encodes a Nudix hydrolase that preferentially degrades diphosphoinositol polyphosphates. *J Virol* **76**, 1415-1421 (2002).

50 Quintas, A. *et al.* Characterization of the African swine fever virus decapping enzyme during infection. *J Virol*, doi:10.1128/jvi.00990-17 (2017).

51 Simon-Mateo, C., Freije, J. M., Andres, G., Lopez-Otin, C. & Vinuela, E. Mapping and sequence of the gene encoding protein p17, a major African swine fever virus structural protein. *Virology* **206**, 1140-1144 (1995).

52 Suarez, C., Gutierrez-Berzal, J., Andres, G., Salas, M. L. & Rodriguez, J. M. African swine fever virus protein p17 is essential for the progression of viral membrane precursors toward icosahedral intermediates. *J Virol* **84**, 7484-7499, doi:10.1128/JVI.00600-10 (2010).

53 Goatley, L. C. & Dixon, L. K. Processing and localization of the african swine fever virus CD2v transmembrane protein. *J Virol* **85**, 3294-3305, doi:10.1128/JVI.01994-10 (2011).

54 Garcia-Beato, R. *et al.* A gene homologous to topoisomerase II in African swine fever virus. *Virology* **188**, 938-947 (1992).

55 Goatley, L. C. *et al.* The African swine fever virus protein j4R binds to the alpha chain of nascent polypeptide-associated complex. *J Virol* **76**, 9991-9999 (2002).

56 Baylis, S. A., Banham, A. H., Vydelingum, S., Dixon, L. K. & Smith, G. L. African swine fever virus encodes a serine protein kinase which is packaged into virions. *J Virol* **67**, 4549-4556 (1993).

57 Freije, J. M., Lain, S., Vinuela, E. & Lopez-Otin, C. Nucleotide sequence of a nucleoside triphosphate phosphohydrolase gene from African swine fever virus. *Virus Res* **30**, 63-72 (1993).

58 Rodriguez, F. *et al.* Characterization and molecular basis of heterogeneity of the African swine fever virus envelope protein p54. *J Virol* **68**, 7244-7252 (1994).

59 Rodriguez, J. M., Garcia-Escudero, R., Salas, M. L. & Andres, G. African swine fever virus structural protein p54 is essential for the recruitment of envelope precursors to assembly sites. *J Virol* **78**, 4299-1313 (2004).

60 Oliveros, M. *et al.* African swine fever virus dUTPase is a highly specific enzyme required for efficient replication in swine macrophages. *J Virol* **73**, 8934-8943 (1999).
61 Rodriguez, I., Nogal, M. L., Redrejo-Rodriguez, M., Bustos, M. J. & Salas, M. L. The African swine fever virus virion membrane protein pE248R is required for virus infectivity and an early postentry event. *J Virol* **83**, 12290-12300, doi:10.1128/JVI.01333-09 (2009).
62 Martinez-Pomares, L., Simon-Mateo, C., Lopez-Otin, C. & Vinuela, E. Characterization of the African swine fever virus structural protein p14.5: a DNA binding protein. *Virology* **229**, 201-211, doi:10.1006/viro.1996.8434 (1997).
63 Andres, G., Garcia-Escudero, R., Vinuela, E., Salas, M. L. & Rodriguez, J. M. African swine fever virus structural protein pE120R is essential for virus transport from assembly sites to plasma membrane but not for infectivity. *J Virol* **75**, 6758-6768, doi:10.1128/JVI.75.15.6758-6768.2001 (2001).
64 Rodriguez, J. M., Salas, M. L. & Vinuela, E. Genes homologous to ubiquitin-conjugating proteins and eukaryotic transcription factor SII in African swine fever virus. *Virology* **186**, 40-52 (1992).
65 de Oliveira, V. L. *et al.* A novel TLR3 inhibitor encoded by African swine fever virus (ASFV). *Arch Virol* **156**, 597-609, doi:10.1007/s00705-010-0894-7 (2011).
66 Henriques, E. S. *et al.* Modeling of the Toll-like receptor 3 and a putative Toll-like receptor 3 antagonist encoded by the African swine fever virus. *Protein science : a publication of the Protein Society* **20**, 247-255, doi:10.1002/pro.554 (2011).
67 Hingamp, P. M., Arnold, J. E., Mayer, R. J. & Dixon, L. K. A ubiquitin conjugating enzyme encoded by African swine fever virus. *EMBO J* **11**, 361-366 (1992).
68 Freitas, F. B., Frouco, G., Martins, C. & Ferreira, F. African swine fever virus encodes for an E2-ubiquitin conjugating enzyme that is mono- and di-ubiquitinated and required for viral replication cycle. *Sci Rep* **8**, 3471, doi:10.1038/s41598-018-21872-2 (2018).

Supplementary information to Figure 2

The following scans show the uncropped immunoblots used in Figure 2. All blots were processed as described in the Methods part and exposed for 8 minutes.

p30 – HEK 293

p30 – Vero

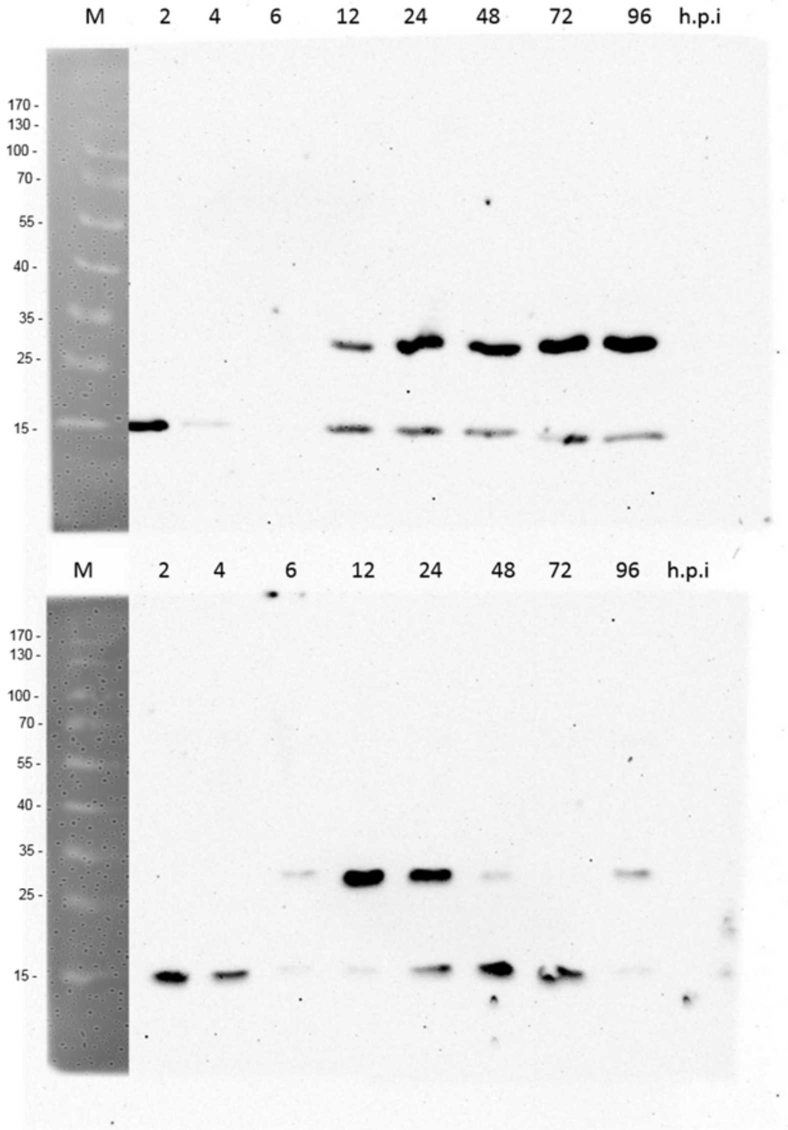

3

p72 – WSL-HP

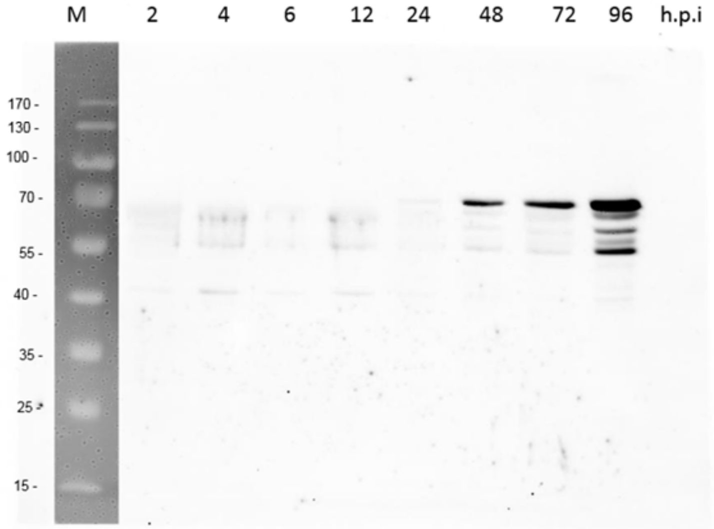

p30 – WSL-HP

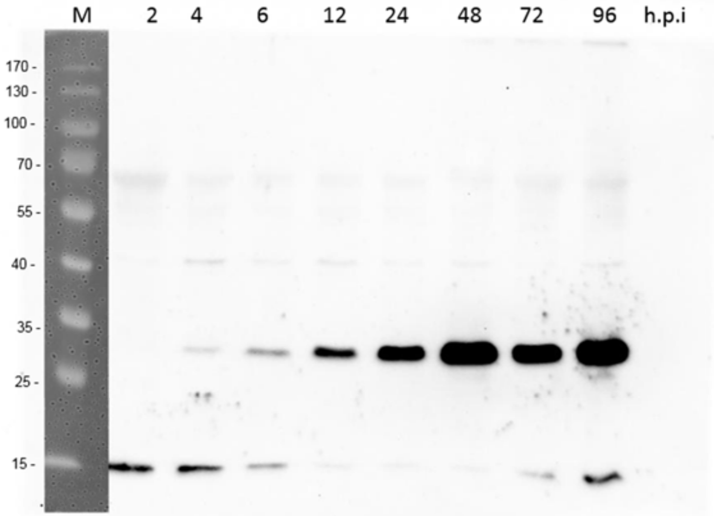

4

5

p72 - Vero

p72 – HEK 293

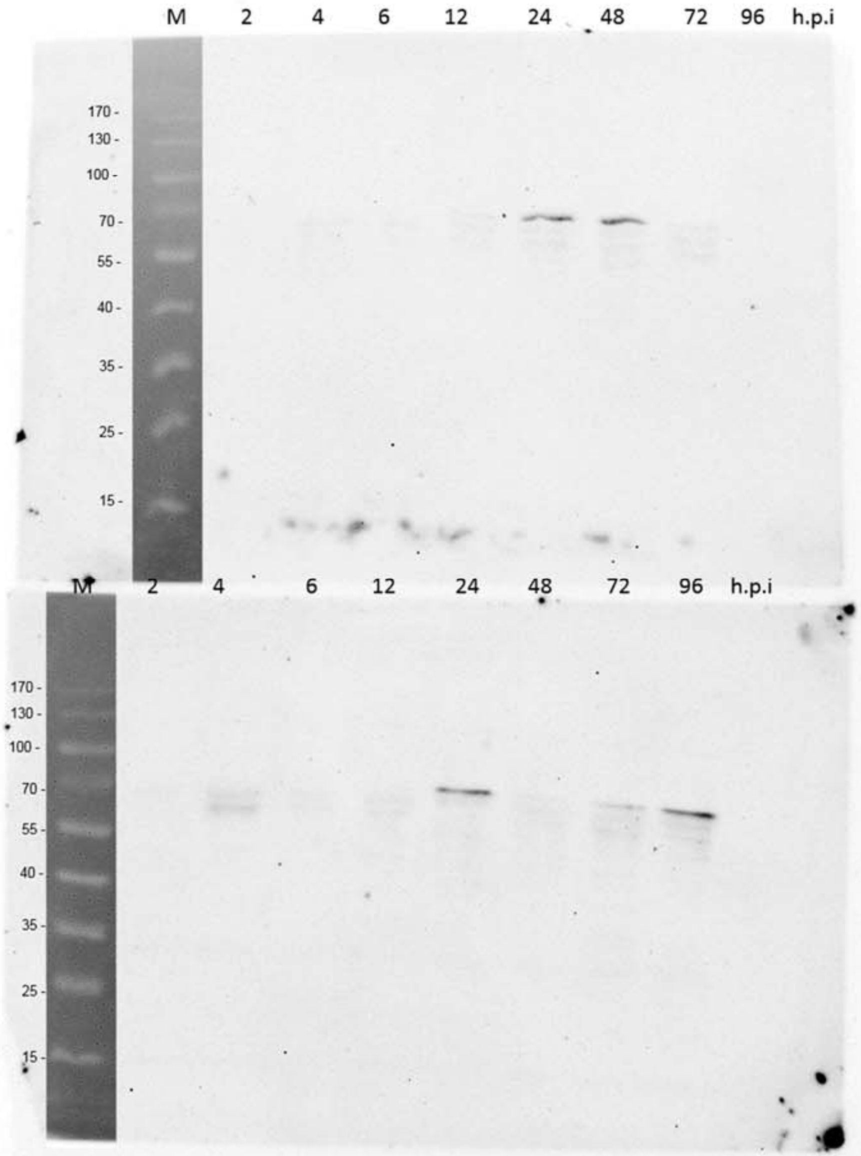

Supplement: Supplementary file 1 — Supplementary Information [file 41598_2018_32985_MOESM1_ESM.pdf]
